# TcVac3 Induced Control of *Trypanosoma cruzi* Infection and Chronic Myocarditis in Mice

**DOI:** 10.1371/journal.pone.0059434

**Published:** 2013-03-26

**Authors:** Shivali Gupta, Nisha Jain Garg

**Affiliations:** 1 Department of Microbiology and Immunology, University of Texas Medical Branch, Galveston, Texas, United States of America; 2 Department of Pathology, University of Texas Medical Branch, Galveston, Texas, United States of America; 3 Faculty of the Institute for Human Infections and Immunity, Center for Tropical Diseases, and Sealy Center for Vaccine Development, University of Texas Medical Branch, Galveston, Texas, United States of America; Federal University of São Paulo, Brazil

## Abstract

We characterized the immune responses elicited by a DNA-prime/MVA-boost vaccine (TcVac3) constituted of antigenic candidates (TcG2 and TcG4), shown to be recognized by B and T cell responses in *Trypanosoma cruzi* (*Tc*) infected multiple hosts. C57BL/6 mice immunized with TcVac3 elicited a strong antigen-specific, high-avidity, trypanolytic antibody response (IgG2b>IgG1); and a robust antigen- and *Tc*-specific CD8^+^T cell response with type-1 cytokine (IFN-γ^+^TNF-α>IL-4^+^IL-10) and cytolytic effector (CD8^+^CD107a^+^IFN-γ^+^Perforin^+^) phenotype. The vaccine-induced effector T cells significantly expanded upon challenge infection and provided >92% control of *T. cruzi*. Co-delivery of IL-12 and GMCSF cytokine adjuvants didn’t enhance the TcVac3-induced resistance to *T. cruzi*. In chronic phase, vaccinated/infected mice exhibited a significant decline (up to 70%) in IFN-γ^+^CD8^+^T cells, a predominance of immunoregulatory IL-10^+^/CD4^+^T and IL10^+^/CD8^+^T cells, and presented undetectable tissue parasitism, inflammatory infiltrate, and fibrosis in vaccinated/infected mice. In comparison, control mice responded to challenge infection by a low antibody response, mixed cytokine profile, and consistent activation of pro-inflammatory CD8^+^T cells associated with parasite persistence and pathologic damage in the heart. We conclude that TcVac3 elicited type-1 effector T cell immunity that effectively controlled *T. cruzi* infection, and subsequently, predominance of anti-inflammatory responses prevented chronic inflammation and myocarditis in chagasic mice.

## Introduction

Chagas disease is prevalent in almost all Latin American countries, including Mexico and Central America. Currently, the World Health Organization estimates that 11–18 million individuals are infected worldwide. Approximately, 13,000 children and adults die annually because of the clinical complications of *T. cruzi*-induced heart disease [1].

A systematic review of the literature suggests that the pathogenesis of the chronic chagasic heart disease is dependent on a low-grade, systemic infection with documented immune-adverse reactions, leading to pathologic tissue injury, and subsequently, to cardiac insufficiency (reviewed in [2]–[3]). An important implication of these studies is that preventing infection or controlling the acute parasite load below a threshold level would be effective in decreasing the tissue damage imposed by multiple pathogenic mechanisms and lead to decreased disease severity, thus, providing an impetus for vaccine development against *T. cruzi*. Extensive research investigating the human and experimental immunity to *T. cruzi* dictates that *Tc-*antigens capable of eliciting lytic antibodies and type 1 T cell responses will be the best choice for prophylactic vaccine against Chagas disease (reviewed in [4]). Accordingly, several antigens (e.g. GP90, TSA-1, cruzipain) have been tested as vaccine candidates in eliciting immunity to *T. cruzi* in small animal models (reviewed in [5]). In parallel, efforts to enhance the protective efficacy of subunit vaccines against *T. cruzi* have included testing the use of adjuvants, e.g. saponin, CpGODN, IL-12 and GMCSF cytokines, [5] attenuated strain of *Salmonella* [6] or adenovirus [7] for antigen delivery, and heterologous prime-boost protocols [8].

We have employed a computational/bioinformatic approach for unbiased screening of the *T. cruzi* genome database, and identified 11 potential candidates. Through rigorous analysis over a period of several years, we considered three candidates (TcG1, TcG2, TcG4) were maximally relevant for vaccine development because these candidates were highly conserved in clinically relevant *T. cruzi* strains, expressed (mRNA/protein) in infective trypomastigote and intracellular amastigote stages of *T. cruzi*, and released during parasite differentiation in host cell cytoplasm, a characteristic required for antigen presentation for cytotoxic T cell activation. Further, TcG1, TcG2 and TcG4 were recognized by IgGs and CD8^+^ T cells in multiple *T. cruzi*-infected hosts [9]–[10], thus, suggesting their potential as vaccine candidates.

In recent studies, we have titrated and shown that immunization of mice with 25-µg of candidate antigens (TcG1, TcG2, or TcG4) by a DNA-prime/DNA-boost approach elicited antibody and CD8^+^ T cells that were further enhanced by co-delivery of IL-12- and GM-CSF-expressing plasmids. The antigen-specific immune responses elicited by the DNA-prime/DNA-boost vaccine provided a degree of control of *T. cruzi* infection but it was not sufficient to prevent chronic myocarditis in mice and dogs [10]. The delivery of candidate antigens by a DNA-prime/protein-boost approach (along with the IL-12 and GM-CSF cytokine adjuvants) was effective in generating type 1 antibody and T cell responses capable of providing ∼90% control of acute parasitemia and tissue parasite burden in infected mice [11]. However, complexity of this vaccine inhibited our ability to move forward with large-scale vaccine design.

Towards our efforts to increase the efficacy and simplify the composition of vaccine against *T. cruzi*, in this study, we delivered the vaccine candidates using a DNA-prime/MVA-boost approach. Modified Vaccinia Ankara (MVA) offers many advantages as a vehicle for delivery of antigens. MVA can accommodate multiple foreign genes in its genome and can be administered by a variety of routes [12]–[13]. MVA and recombinant MVA (rMVA) have excellent safety records in immune-compromised mice, monkeys as well as in humans, and shown to generate cellular and humoral responses to a variety of foreign antigens (reviewed in [14]). We found that thet delivery of TcG2 or TcG4 by DNA-prime/MVA-boost approach elicited potent antigen-specific antibody and T cell response [15].

Build on this background work, in this study; we have tested the protective efficacy of TcVac3 constituted of TcG2 and TcG4 candidates, delivered by the DNA/MVA approach. We chose to employ heterologous DNA/MVA approach because other studies have indicated that delivery of antigen (e.g. glycoprotein D of HSV-2, ME-TRAP of *Plasmodium falciparum*, 85A of *Mycobacterium tuberculosis*) by a DNA-prime/MVA-boost approach resulted in a greater level of antigen-specific antibody, T cell, and cytokine responses than was observed by homologous prime/boost approach [16]–[17]. We also tested whether DNA-MVA approach will increase the vaccine efficacy sufficiently to omit the need for IL-12 and GM-CSF adjuvants. We discuss the function of vaccine-induced antibody and T cell responses against *T. cruzi* in providing protection from acute parasitemia, and chronic parasite persistence and immunopathology in chagasic mice.

## Materials and Methods

### Parasites and Mice

*T. cruzi* trypomastigotes (Sylvio X10/4 strain) were maintained and propagated by continuous *in vitro* passage in C2C12 cells. C57BL/6 female mice (6-to-8 weeks old) were obtained from Harlan Labs (Indianapolis, IN). Animal experiments were performed according to the National Institutes of Health Guide for Care and Use of Experimental Animals and approved by the UTMB Animal Care and Use Committee.

### *T. cruzi* Genes and Generation of Recombinant Plasmids for Vaccination

The cDNAs for TcG2 and TcG4 (SylvioX10 isolate, Genbank: AY727915 and AY727917, respectively) were cloned in eukaryotic expression plasmid pCDNA3.1 [18]. Plasmids encoding IL-12 (pcDNA3.msp35 and pcDNA3.msp40) and GM-CSF (pCMVI.GM-CSF) have been previously described [17]. All recombinant plasmids were transformed into *E. coli* DH5-α competent cells, grown in L-broth containing 100-µg/ml ampicillin, and purified using the Endo-free Maxi Prep kit (Qiagen, Chatsworth, CA).

### Generation of Recombinant MVA

The pLW44 vector consists of a green fluorescent protein (GFP) and multiple cloning site (MCS) cassette flanked by a pair of MVA genomic sequences which allows homologous recombination and incorporation of both GFP and the gene of interest into the deletion III locus of the wild-type MVA (wtMVA) genome. We sub-cloned *TcG2* and *TcG4* at the Xma1/Sbf1 sites of pLW44, and sequenced the recombinant plasmids at the Molecular Genomics Core Facility at the UTMB.

BHK-21 cells at 70% confluency (six-well plate) were infected with wtMVA (MOI of 0.05) for one h, and then transfected with pLW44.TcG2 or pLW44.TcG4 (2-µg DNA) mixed with Lipofectamine 2000 (Invitrogen, Grand Island, NY) and cells cultured for 48 h. Cell lysates were added at 10-fold dilutions to new BHK-21 cell monolayers in six-well plates, and after 1 h of infection, cells were overlaid with 2% methylcellulose (Sigma, St. Louis, MO), and incubated as above. Two days later, at least three GFP^+^ fluorescent plaques were picked for each rMVA. The plaque purification procedure was repeated 4–6 times to ensure removal of wtMVA contamination.

For amplification of rMVAs, BHK-21 cell monolayers were propagated in T-150 tissue culture flasks and inoculated with rMVA (MOI: 0.5). At 72 h post-incubation, cells were pelleted, lysed in 10 mM Tris–HCl (pH 9) using a dounce homogenizer, and centrifuged at 500×g. The recombinant virus containing supernatants were purified twice on a 36% sucrose cushion in a swing bucket rotor (SW-41 followed by SW-28) by centrifugation at 13,500 rpm, 4°C for 60–80 min. The viral pellets were stored in 1 mM Tris–HCl (pH 9) at −80°C [15].

### Immunization and Challenge Infection

C57BL/6 mice were injected with antigen-encoding plasmids (pCDNA3.TcG2 and pCDNA3.TcG4) with or without IL-12- (pCDNA3.msp35, pCDNA3.msp40) and GM-CSF (pCMV.GMCSF)-encoding plasmids (25-µg each plasmid DNA/mouse, i.m.,1^st^-dose).Three weeks later, mice were given booster vaccine (2^nd^-dose) constituted of rMVA.TcG2 and rMVA.TcG4 (10^6^-pfu each/mouse, i.d.). Mice injected with empty vectors were used as controls. Two-weeks after the last immunization, mice were challenged with *T. cruzi* (10,000 trypomastigotes/mouse, i.p.). Mice were sacrificed at day 30- and 120-post-infection (pi) corresponding to the acute phase of peak parasitemia and the chronic phase of disease development, respectively. Sera and tissue samples were stored at 4°C and −80°C, respectively.

### Recombinant Proteins

The cDNAs for *TcG2* and *TcG4* were cloned in-frame with a C-terminal His-tag in to pET-22b plasmid (Novagen, Gibbstown, NJ). All cloned sequences were confirmed by restriction digestion and sequencing at the Molecular Genomics Core Facility at UTMB. Plasmids were transformed in *BL21* (DE3) pLysS competent cells, and recombinant proteins purified using the poly-histidine fusion peptide-metal chelation chromatography system [11].

### Antibody Levels, Avidity and Trypanolytic Activity

*T. cruzi* lysate (TcTL, 5×10^5^ parasites’ equivalent/well) or recombinant TcG2 and TcG4 proteins (1-µg/well) were used to capture the sera levels of *Tc*- and antigen-specific antibodies in an ELISA [9]. To identify the antibody sub-types, plates were coated as above, blocked with PBS/5% non-fat dry milk (NFDM, Bio-Rad), and then sequentially incubated with sera samples (1∶100–1∶1000 dilution, 100-µl/well) for 2 h, biotin-conjugated goat anti-mouse Ig subtypes (IgG1, IgG2a or IgG2b) for 2 h, and streptavidin-horseradish peroxidase conjugate for 30 min. All antibodies and conjugates were from Southern Biotech, and used at a 1∶5000 dilution in PBS-0.01% Tween-20/0.5% NFDM (100-µl/well). Color was developed with Sure Blue 3,3′,5,5′-tetramethylbenzidine substrate (K&P Labs, Gaithersburg, MD), and monitored at 450 nm using a SpectraMax M5 microplate reader [11].

To examine antigen-avidity index, plates were coated with antigens, blocked with 5% NFDM, and then sequentially incubated with sera samples (1∶100–1∶1000 dilution in PBS/1% NFDM) for 1 h, 6 M urea for 30 min, and HRP-conjugated secondary antibody for 1 h. A colorimetric reaction was performed as above (% Avidity: [O.D. with urea/O.D. without urea treatment] × 100) [19]–[20].

To evaluate trypanolytic activity of vaccine-induced antibodies, *T. cruzi* trypomastigotes (5×10^4^/25-µl) were incubated for 4 h at 37°C with 25-µl each of sera samples (1∶8–1∶80 dilution) and human complement (Sigma), and percentage of live parasites monitored by MTT assay [21]. Controls included *Tc* incubated with pre-immune sera, complement alone, or immune sera with heat-inactivated complement.

### Lymphocytes’ Activation and Proliferation, Intracellular Cytokines, and Cytokine Release

Single-cell splenocyte preparation (10^6^-cells/ml RPMI-5% FBS) were distributed in 24-well plates, and incubated in presence of Con A (5 µg/ml), recombinant proteins (10-µg/ml), or TcTL (25-µg/ml) for 8–48 h at 37°C/5% CO_2_. Culture supernatants were collected for the measurement of IL-4, IL-10, IFN-γ, and TNF-α cytokines using optEIA^tm^ ELISA kits (Pharmingen, San Diego, CA). Splenocytes (stimulated or un-stimulated) were labeled for 30-min on ice with PE-Cy7 conjugated anti-CD3 (binds all T cells), FITC-conjugated anti-CD8 and PE-conjugated anti-CD4 antibodies (0.5–1 µg/100 µl, e-Biosciences, San Diego, CA). Cells were fixed with 2% paraformaldehyde, and analyzed on a LSRII Fortessa Cell Analyzer (BD Biosciences, San Jose, CA).

To monitor the contribution of T cell subsets to cytokine response, splenocytes were *in vitro* stimulated as above, except that brefeldin A (10-µg/ml; Sigma) or monensin (5-µg/ml) was added in the final 6 h to prevent protein secretion. Cells were labeled with anti-CD4 and anti-CD8 antibodies, fixed with 4% paraformaldehyde, resuspended in 100-µl permeabilization buffer (0.1% saponin/1% FBS in PBS) and then utilized for intracellular staining with APC-anti-IL-4, PerCPCy5.5-anti-IL-10, e-Fluor-anti-IFN-γ, Cy5-anti-TNF-α and PerCP-Cy5.5-anti-Ki67 antibodies (0.5–2-µg/100-µl, e-Biosciences). In some experiments, splenocytes were also incubated with APC-anti-perforin or Alexa-Fluor 488-anti-CD107 antibodies to determine the cytolytic activity of the activated/proliferating T cell subpopulations. Cells stained with isotype-matched IgGs were used as controls. Samples were visualized on a LSRII Fortessa Cell Analyzer by six-color flow cytometry, acquiring 30–50,000 events in a live lymphocyte gate, and further analysis performed using FlowJo software (version 7.6.5, Tree-Star, San Carlo, CA).

### Blood and Tissue Parasite Burden

Blood DNA was isolated with QiAamp Blood DNA mini kit (Qiagen, Chatsworth, CA). Skeletal muscle and heart tissues (50-mg) were subjected to proteinase K lysis, and total DNA was purified by phenol/chloroform extraction and ethanol precipitation. Total DNA (50-ng) was used as a template, and real-time PCR performed on an iCycler thermal cycler with SYBR Green Supermix (Bio-Rad) and Tc18S-specific oligonucleotides. Data were normalized to murine-GAPDH, and fold change in *Tc* burden calculated as 2^−ΔCt^, where ΔC_t_ represents the C_t_ (infected) − C_t_ (control) [22].

### Histopathology

Paraffin-embedded tissue-sections (5-micron) were stained with hematoxylin and eosin, and scored as previously described [23]. The presence of inflammatory cells was scored as (0) - absent/none, (1) - focal or mild with ≤1 foci, (2) - moderate with ≥2 inflammatory foci, (3) - extensive with generalized coalescing of inflammatory foci or disseminated inflammation (4) - severe with diffused inflammation, interstitial edema, and loss of tissue integrity. The foci of pseudocysts (*Tc* nests) were scored as (0) absent, (1) 0–1 foci, (2) 1–5 foci, and (3)>5 foci. Masson’s Trichrome-stained tissue-sections were assessed for fibrosis (blue-colored collagen area) and H&E stained tissues for inflammation as a percentage of the total myocardial area using Simple PCI software (version 6.0; Compix, Sewickley, PA) connected to an Olympus polarizing microscope system (Center Valley, PA) (n = 4/group, 10-slides/tissue).

### Statistical Analysis

Data are expressed as mean ± SD (n = 8/group, triplicate observations per experiment). Data were analyzed by the Student *t* test (comparison of two-groups) and 1-way analysis of variance (ANOVA) using Graph Pad InStat ver.3 software. Significance is shown by *p<0.05, **p<0.01, ***p<0.001 (vaccinated–versus-control).

## Results

### TcVac3 Induced High Avidity Lytic Antibodies, Type 1 Cytokine Response and Effector CD8^+^T Cell Response

**Figure 1:**
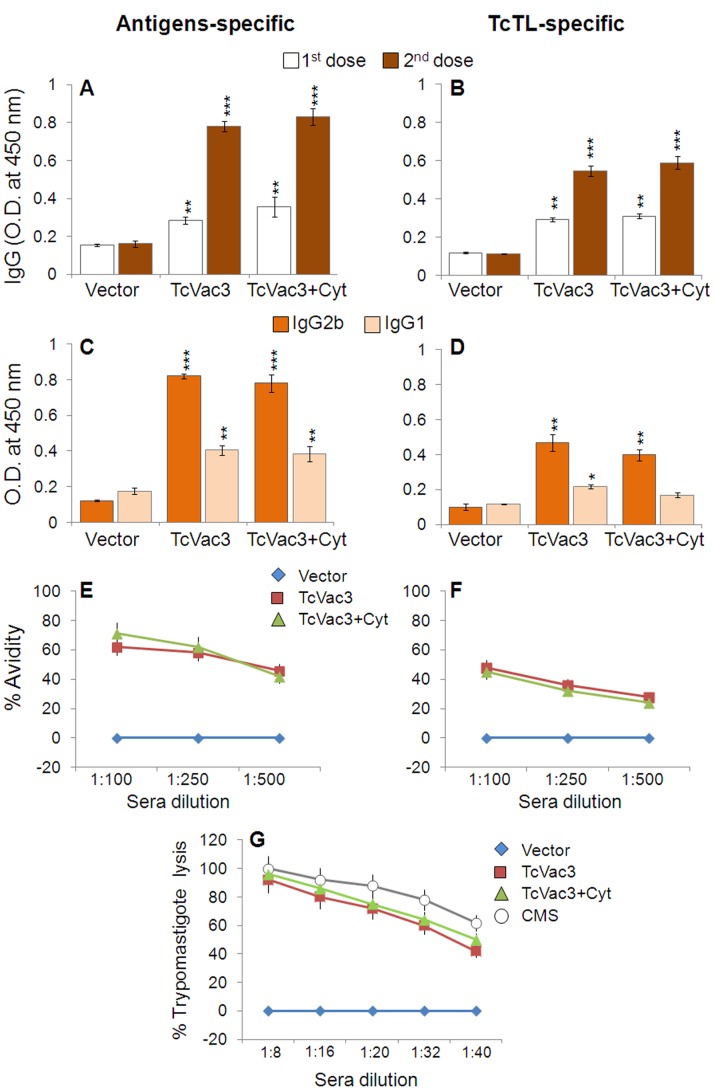
DNA-prime/MVA-boost delivery of *T. cruzi* candidate antigens induced Th1 polarized, high-avidity, lytic antibody response in mice. C57BL/6 mice were injected with empty vector or TcVac3 (± cytokine adjuvants), as detailed in Materials and Methods, and sera samples were obtained two-weeks after each immunization. (**A–D**) Sera levels of IgG (A&B) and IgG2b/IgG1 subtypes (C&D) were determined by an ELISA using recombinant antigens (A&C) or *T. cruzi* lysate (TcTL, B&D) to capture the antibodies. (**E&F**) Shown is antigen-specific (E) and parasite-specific (F) percent avidity of sera antibodies induced in vaccinated mice. (**G**) Tryapanolyitc activity of sera antibodies induced in vaccinated mice. The data presented in C–G was derived from sera samples obtained 2-weeks after second immunization. CMS: Chronically-infected mouse serum. In all figures, data are presented as mean ± SD, and representative of three independent experiments (n≥8, *p<0.05, **p<0.01, ***p<0.001, vaccinated-versus-controls or vaccinated/infected-versus-infected controls).

The antigen*-* and *Tc*-specific IgGs were detectable after the 1^st^ vaccine dose, and increased significantly following rMVA immunization (Fig. 1A&B, sera dilution: 1∶100, p<0.01). TcVac3-induced IgGs were primarily constituted of IgG2b subtype (IgG2b/IgG1>1, Fig. 1C&D, *p*<0.01) and IgG2a antibodies were not detectable (data not shown). Normal mice and mice injected with vector only exhibited no *Tc*- and antigen-specific antibody response (Fig. 1A–D). TcVac3-induced antibodies exhibited a substantial level of antigen (range: 68–75%) and *T. cruzi* (range: 48–50%) binding capacity at sera dilution of 1∶100 that declined in a linear fashion with further sera dilutions (Fig. 1E&F, *p*<0.001). Further, TcVac3-induced antibodies exhibited 92–96% complement-dependent trypanolytic efficiency at sera dilution of 1∶8 that was comparable to that noted in chronically-infected mice exposed to multiple *Tc* antigens (Fig. 1G). A linear decline in trypanolytic activity of vaccine-induced antibodies with further dilution of sera samples from vaccinated mice was similar to that noted for chronic mouse sera (Fig. 1G). No lysis was observed when trypomastigotes were incubated with heat-inactivated immune serum alone, complement alone, or heat-inactivated immune serum plus heat-inactivated complement (data not shown). No lytic activity was detected when trypomastigotes were incubated with sera from control mice in presence or absence of complement, thus confirming the specificity of the lytic activity of the vaccine-induced antibodies. Together, these data demonstrate that TcVac3 elicited a strong antigen-specific Th1 type antibody response that exhibited a high degree of antigen-specific avidity and *Tc*-specific lytic activity. Co-delivery of cytokine adjuvants had no significant enhancing effect on TcVac3-induced antigen- and *Tc*-specific antibodies’ levels, avidity and lytic activity (Fig. 1).

**Figure 2:**
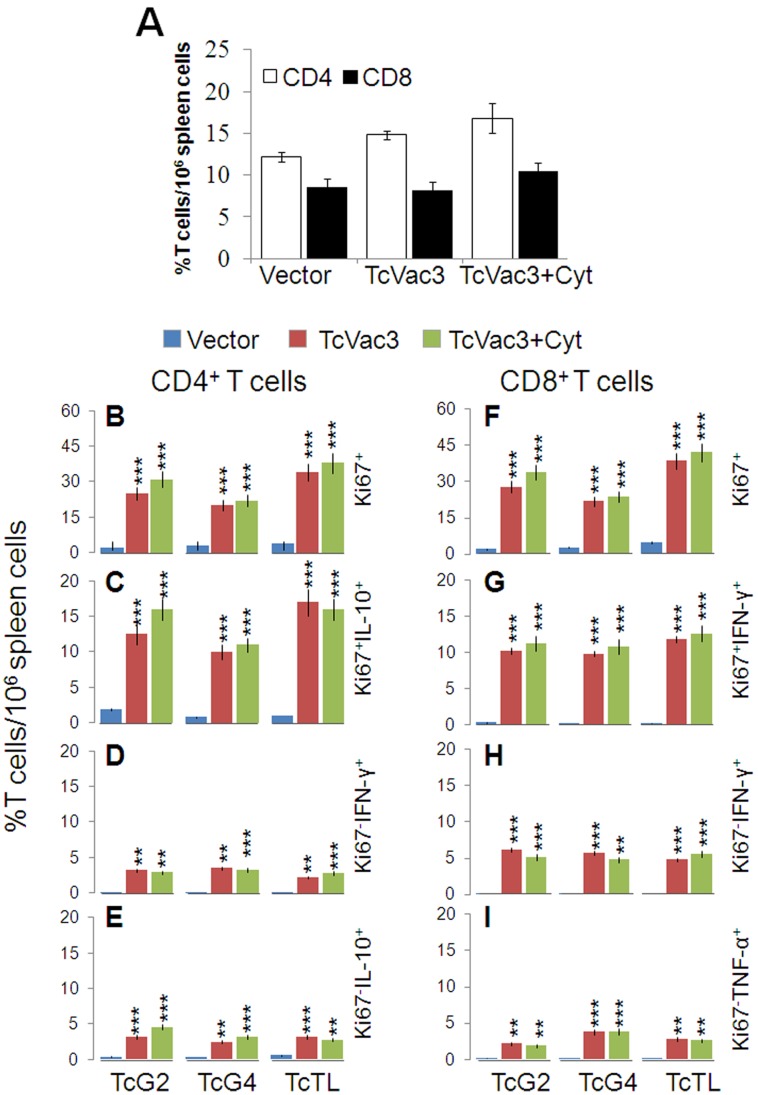
Splenic T cells cytokine profile elicited by TcVac3 immunization. Mice were immunized as in Fig. 1, and harvested two-weeks after last immunization. (**A**) Splenocytes were incubated with PE-conjugated anti-CD4 and FITC-conjugated anti-CD8 antibodies, and T cell subsets monitored by flow cytometry. (**B–I**) Splenocytes were *in vitro* stimulated for 48 h with recombinant antigens or TcTL. Shown are the mean percentage of PE^+^CD4^+^T cells that were Ki67^+^ (PerCPCy5.5, B) and IL-10^+^ (e-Fluor, C), and Ki67^−^CD4^+^T cells that were IFN-γ^+^ (e-Fluor, D) and IL-10^+^ (APC, E). Shown are the mean percentage of FITC^+^CD8^+^T cells that were Ki67^+^ (F) and IFN-γ^+^ (G), and Ki67^−^CD8^+^T cells that were IFN-γ^+^ (H) and TNF-α^+^ (Cy5, I).

To examine the TcVac3-primed T cell profile, splenocytes (2-weeks after 2^nd^-dose) were *in vitro* stimulated with recombinant antigens or TcTL, and cell free supernatants were utilized for the measurement of cytokines by ELISA. Splenocytes were gated for CD4^+^T and CD8^+^T cells and then analyzed for proliferative capacity (Ki67^+^), intracellular cytokine profile (IFN-γ, TNF-α, IL-4, IL-10) and lytic activity by flow cytometry. We noted a substantial IFN-γ (192–287-pg/ml), TNF-α (112–212-pg/ml), and IL-10 (104–148-pg/ml) release in the supernatant of *in vitro* stimulated splenocytes of TcVac3-immunized mice (Table 1, *p*<0.05). Incubation with Con A resulted in splenic activation of IFN-γ, TNF-α, IL-4 and IL-10 cytokines (range: 650–1800-pg/ml), irrespective of vaccination status (data not shown). The *in vivo* percentages of CD4^+^T (range: 14.8–16.8%) and CD8^+^T (range: 8.2–10.5%) cells was comparable in vaccinated and control mice (Fig. 2A). Yet, splenic T cells from TcVac3-immunized mice exhibited a significant antigen- and *Tc*-specific proliferation (CD4^+^Ki67^+^: 20–38%; CD8^+^Ki67^+^: 24–42%, Figs. 2B&F, p<0.001). A majority of the TcVac3-induced Ki67^+^CD4^+^T cells were IL-10^+^ (Fig. 2C, p<0.001), and the non-proliferating (Ki67^−^) CD4^+^T cells were low cytokine producers (Fig. 2D&E). Further, a significant number of the TcVac3-induced proliferating (Ki67^+^) and non-proliferating (Ki67^−^) CD8^+^T cells produced IFN-γ and low levels of TNF-α in an antigen-specific manner (Fig. 2G-I, p<0.01–0.001). The TNF-α^+^CD4^+^, IL-4^+^CD8^+^ and IL-10^+^CD8^+^ T cells were not detectable in TcVac3-immunized mice (data not shown). Co-delivery of cytokine adjuvants did not alter the TcVac3-induced T cell responses, and no *Tc*-specific expansion of CD4^+^T or CD8^+^T cells was observed in control mice given empty vector only (Fig. 2). Together, these results suggested that TcVac3 primed a robust antigen-specific type 1 CD8^+^T cell response and co-delivery of cytokine adjuvants was dispensable for the induction of antigen-specific T cell responses in vaccinated mice.

**Table 1:** Splenocytes’ cytokine profile in mice after vaccination with TcVac3 and challenge infection with *T. cruzi.*

In vitro stimul-ation	Day 14 post-vaccination				Day 30 post-infection				Day 120 post-infection		
	Control	TcVac3	TcVac3+Cytokines		Control	TcVac3	TcVac3+Cytokines		Control	TcVac3	TcVac3+Cyt
	**IFN-γ (pg/ml)**										
None	5±0.86	10±2	ND	14±2		15±3		10±1.8	11±2	ND	12±4
TcG2	10±2	192±20***	205±26***	260±11		1480±28***		1515±42***	100±8	275±10**	515±18***^,###^
TcG4	12±2	260±15***	287±12***^,#^	350±10		1622±26***		1610±22***	230±11	265±11	610±32***^,###^
TcTL	6±1	198±6***	225±22***^,#^	1050±9		1820±45***		1845±66***	1002±6	820±20**	845±28***
	**TNF-α (pg/ml)**										
None	nd	8±2	12±2.8	12±3		13±3	16±3		15±4	12±3	10±1
TcG2	11±2	162±18***	212±15***^,#^	255±16		754±16***	765±15***		235±12	154±16**	195±11**^,##^
TcG4	10±3	160±12***	205±13***^,#^	250±14		708±18***	770±12***^,#^		215±14	235±12	208±10^#^
TcTL	8±2	115±8***	112±14***	545±21		815±20***	842±22***		505±20	295±11***	242±14***^,##^
	**IL-4 (pg/ml)**										
None				11±2		24±3	26±6		8±1	ND	ND
TcG2	Undetectable			22±3		112±18***	155±20***^,##^		26±3	223±16***	245±18***
TcG4				20±2		120±14***	122±18***		23±2	110±14***	221±17***
TcTL				45±6		123±11***	125±15***		110±16	320±18***	355±22***
	**IL-10 (pg/ml)**										
None	4±0.9	12±2	10±3	12±2		15±3	18±4		10±2	12±3	15±4
TcG2	8±2	148±11***	135±10***	16±3		146±13***	276±21***		18±3	146±13***	676±21***
TcG4	10±4	125±15***	115±8***	22±3		225±11***	280±26***		25±3	325±11***	380±16***
TcTL	14±4	112±14***	104±10***	425±36		272±22***	280±23***		320±22	380±22*	680±23***^,##^

**Figure 3:**
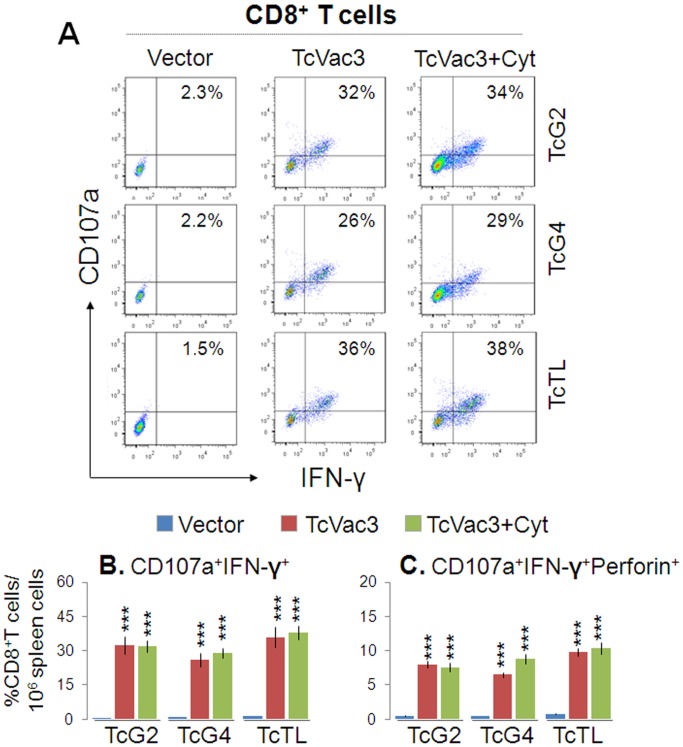
Cytolytic functional profile of TcVac3-induced splenic CD8^+^T cells. Mice were immunized as above, and splenocytes harvested two-weeks after last dose were *in vitro* stimulated with recombinant antigens or TcTL. (**A**) Shown are representative quadrant images acquired by flow cytometry of PE^+^CD8^+^T cells exhibiting CD107a^+^ (Alexa Fluor 488) and IFN-γ^+^ (e-Fluor) phenotype. (**B&C**) Percentage of antigen-specific and *Tc*-specific CD8^+^IFN-γ^+^T cells that were CD107a^+^ (B) and CD107a^+^Perforin^+^ (C), acquired from flow cytometry analysis.

Transport of CD107a and CD107b integral membrane proteins to the plasma membrane of effector T cells is required for a) the cytolytic activity mediated by perforin and granzymes and b) the release of IFN-γ which exerts pleiotropic effects to suppress intracellular pathogens. Flow cytometry studies showed TcVac3 induced a high frequency of antigen- (26–34%) and *Tc*- (36%) specific CD107a^+^IFN-γ^+^CD8^+^T cells (Fig. 3A&B, *p*<0.001), and up to 60% of these cells (6.6–9.8% of the total CD8^+^T cells) were perforin^+^ (Fig. 3C, *p*<0.001). Cytokine adjuvants did not enhance the TcVac3-induced antigen- or *Tc*-specific functional CD8^+^T cells, and no expansion of functional CD8^+^T cells was observed in control mice given vector only (Fig. 3). Taken together, the results presented in Figs. 2&3, and Table 1 suggested that TcVac3 elicited both CD4^+^ and CD8^+^ T cell proliferation. TcVac3-induced CD8^+^T cells were predominantly IFN-γ^+^ with cytolytic capacity, and, thus, had a potential to act as effector T cells against *T. cruzi*. The co-delivery of cytokine adjuvants with TcVac3 had no clear additive effect in enhancing the antigen-specific functional CD4^+^ and CD8^+^ T cell profile.

### Expansion of TcVac3-primed Type 1 Antibody Response After Challenge Infection

**Figure 4:**
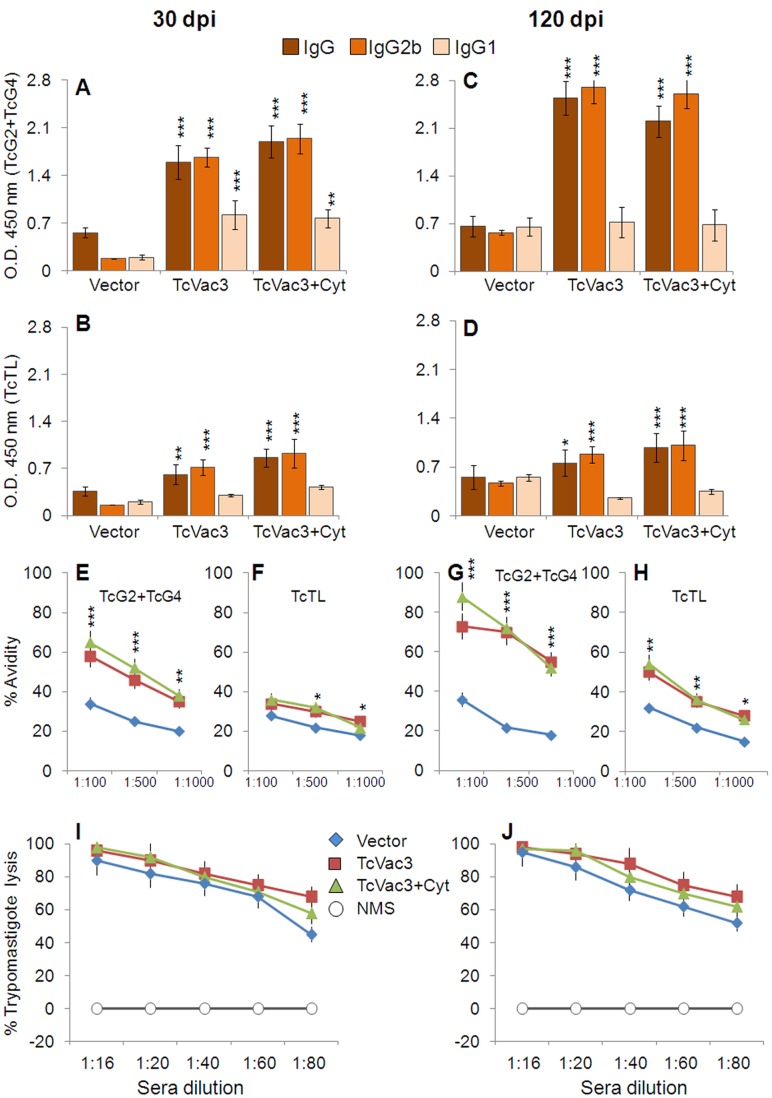
TcVac3-immunized mice responded to challenge *T. cruzi* infection by a strong expansion of parasite- and antigen-specific antibody response. Mice were immunized as in Fig. 1, and challenged with *T. cruzi* (10,000/mouse). The sera levels of antigen-specific (**A&C**) and TcTL-specific (**B&D**) IgG, IgG2b and IgG1 antibodies were measured at day 30 (A&B) and 120 (C&D) post-infection by an ELISA. (**E–H**). Shown are antigen-specific (E&G) and TcTL-specific (F&H) percent avidity of the antibodies induced in vaccinated/infected mice at day 30 (E&F) and 120 (G&H) pi. (**I&J**) Trypanolytic activity of sera antibodies induced in vaccinated/infected mice at day 30 (I) and 120 (J) pi.

Sera samples were collected at day 30 and 120 pi corresponding to acute and chronic stages of infection and disease development to evaluate the quality and quantity of antibody response elicited in vaccinated mice. TcVac3-immunized mice exhibited a potent expansion of *Tc*- and antigen-specific IgG response upon challenge infection, evidenced by ∼10–30-fold increase in vaccine-induced antibody response (sera dilution 1∶1000, compare Fig. 4A&B with Fig. 1A&B and). *Tc*- and antigen-specific IgG, IgG2b titers were 2–4-fold and ∼6-fold higher in TcVac3-immunized mice as compared to that noted in controls at day 30 and 120 pi ((IgG2b>IgG1, Fig. 4A–D, *p*<0.001). Further, sera antibodies in TcVac3-immunized mice exhibited antigen- and TcTL-binding capacity of 32–65% and 60–80% at day 30 and 120 pi, respectively, at sera dilution of 1∶100, that was 1.5–2-fold higher than that noted in infected controls (Fig. 4E–H, *p*<0.05). We also noted the sera antibodies from vaccinated/infected mice exhibited 90–92% and 94–96% complement-dependent trypanolytic efficiency, respectively, at sera dilution of 1∶16 (Fig. 4I&J). Co-delivery of cytokine adjuvants did not enhance the hosts’ capacity to respond to challenge infection by more potent expansion of *Tc*-specific antibodies than was observed in TcVac3-immunized/infected mice (Fig. 4). Together, these results suggested that TcVac3-immunized mice responded to *T. cruzi* by a rapid expansion of *Tc*- and antigen-specific, high avidity, type 1 antibody response, and this response increased with time, likely as an outcome of progressive affinity maturation and antigen-driven B-cell selection.

### Predominant Expansion of Type 1 Cytokines and CD8^+^T Cells in Response to Challenge Infection in TcVac3-immunized Mice

**Figure 5:**
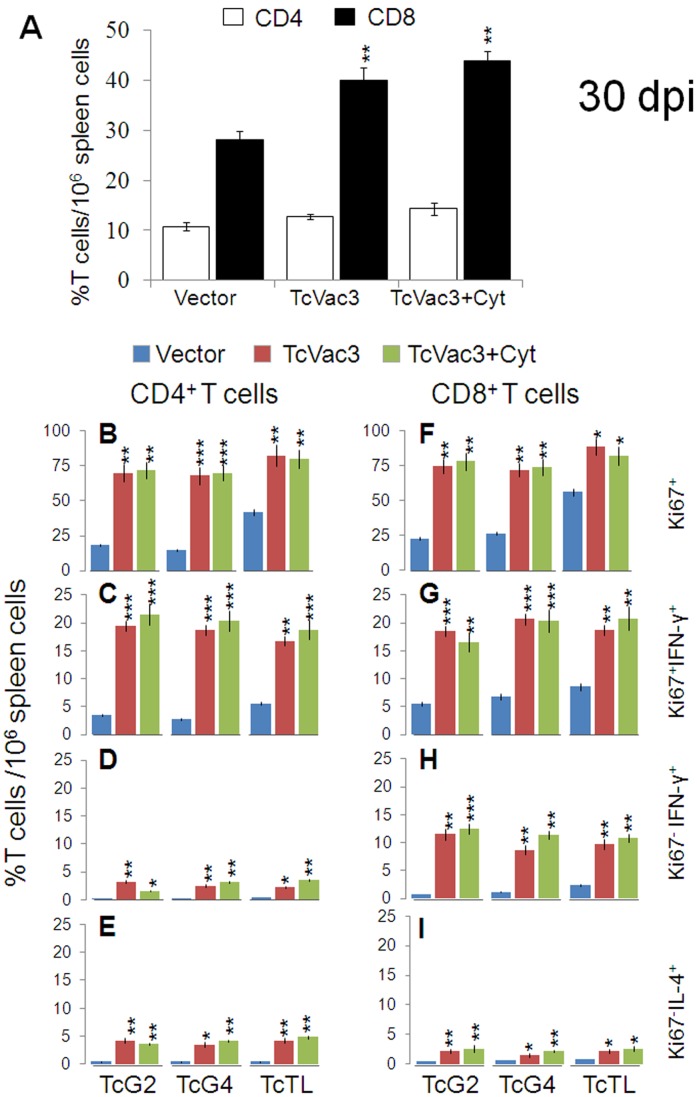
Functional profile of TcVac3-induced T cells in response to challenge infection by *T. cruzi*. Mice were injected with empty vector or TcVac3 (± cytokine adjuvants), infected with *T. cruzi,* and harvested at day 30 post-infection. (**A**) Splenic T cell profile was determined by flow cytometry after staining with FITC-conjugated anti-CD8 and PE-conjugated anti-CD4 antibodies. (**B–I**) Splenocytes were *in vitro* stimulated with recombinant antigens or TcTL for 48 h, and incubated with fluorescent-conjugated antibodies as described in Fig. 2. Shown are the CD4^+^ (**B–E**) and CD8^+^ (**F–I**) proliferating (Ki67^+^, B,C,F,G), and non-proliferating (Ki67^−^, D,E,H,I) T cell subsets and their intracellular cytokine (IFN-γ: C,D,G,H; IL-4: E&I*)* profile, measured by flow cytometry.

Next, we determined if vaccinated mice were better equipped in responding to *T. cruzi* infection by expansion of functional CD4^+^ and/or CD8^+^ T cells. TcVac3-immunized and control mice exhibited no change in CD4^+^T cells, and 4–5-fold and ∼3-fold increase in splenic CD8^+^T cells, respectively, in response to acute infection (day 30 pi, Fig. 5A). Splenic cells from infected mice, irrespective of the vaccination status, responded to *in vitro* stimulation with recombinant antigens or TcTL by a notable, predominantly type 1 cytokines (IFN-γ+TNF-α>IL-10+IL-4) production (Table 1). The splenic IFN-γ and TNF-α levels were significantly higher in TcVac3-immunized/infected mice as compared to that observed in infected controls (p<0.05–0.001). Flow cytometry studies showed 68–84% of the CD4^+^T cells from TcVac3-immunized/infected mice proliferated in an antigen- and TcTL-specific manner (Fig. 5B), and a significant proportion of these cells were IFN-γ^+^ (Fig. 5C). Non-proliferating Ki67^−^CD4^+^T cells exhibited a low IFN-γ^+^ (2–4%) or IL-4^+^ (3.5–5%) phenotype (Fig. 5D&E). Infected control mice, with the exception of IL-10^+^T cells (range: 1.6–5.5%), exhibited a remarkably low CD4^+^T cell proliferation or cytokine production in response to acute *T. cruzi* infection (Fig. 5B–E, Table 2).

**Table 2:** Splenic T cell functional profile in TcVac3-immunized mice infected with *T. cruzi.*

In vitro stimulation	Control	TcVac3	TcVac3+Cytokines
	Day 30 pi, CD4+Ki67-IL-10+		
TcG2	2.8±1.1	1.2±0.8	2.6±0.8
TcG4	1.6±.6	1.5±0.5	1.2±0.6
TcTL	5.5±1.5	2.6±0.6**	2.8±0.8**
	Day 30 pi, CD8+Ki67+TNF-α+		
TcG2	2.5±0.7	14.5±2.6***	12.5±2.6***
TcG4	3.8±1	12.7±2.8***	16.4±3.1***
TcTL	6.6±1.6	16.7±3.5***	15.8±2.8***
	Day 30 pi, CD8+Ki67-TNF-α+		
TcG2	0.8±0.2	6.8±1***	6.5±1.5***
TcG4	1.2±0.6	4.7±0.9**	5.4±0.8***
TcTL	2.4±0.8	4.8±0.6**	5.8±1***
	Day 120 pi, CD8+Ki67+TNF-α+		
TcG2	1.8±0.8	2.6±0.9	2.2±0.2
TcG4	2.6±0.9	2.8±0.8	1.8±0.3
TcTL	5.6±1.2	3.1±0.9*	3.8±1.4*

Mice were immunized with empty vector or TcVac3 (± cytokine adjuvants), and infected with *T. cruzi*, as detailed in Materials and Methods. Splenocytes were obtained 2-weeks after vaccination or at day 30 (acute) and 120 (chronic) post-infection, and *in vitro* stimulated for 48 h with TcG2 and TcG4 recombinant antigens or *T. cruzi* lysate (TcTL). The cell free supernatants were used to measure IFN-γ, TNF-α, IL-4, and IL-10 levels by an ELISA (A). Cells were labeled with appropriate fluorescent-tagged antibodies, and flow cytometry was performed to gate for proliferating (Ki67+) and non-proliferating (Ki67-) CD8+ T cells producing cytokines (B). Data (mean ± SD) are representative of three independent experiments (n = 4/group). ND = Not detectable. Statistical analysis was done using ANOVA with post hoc Tukey’s test. The level of significance between control versus vaccinated (TcVac3 or TcVac3+cytokine adjuvants) is shown by *and between TcVac3 versus TcVac3+cytokine adjuvants by # (*^,#^p<0.05; **^,##^p<0.01; ***^,###^p<0.000).

When analyzing CD8^+^T cell profile of TcVac3-immunized/acutely-infected mice, we noted 72–88% of the CD8^+^T cells were Ki67^+^ (Fig. 5F), and up to 20% of these cells were IFNγ^+^ or TNF-α^+^ (Fig. 5G, Table 2), indicating a prolific antigen- and *Tc*-specific proliferation and activation. The non-proliferating (Ki67^−^) CD8^+^T cells exhibited a moderate TNF-α^+^and IFN-γ^+^, and a low IL-4^+^ phenotype (Fig. 5H&I, Table 2). No IL-10 production was observed by either proliferating or non-proliferating CD8^+^T cell types. The TcVac3-dependent expansion of CD4^+^ and CD8^+^ T cell response to challenge infection was not significantly altered when cytokine adjuvants were co-delivered with the 1^st^-dose of vaccine (Fig. 5). Infected control mice, in comparison to TcVac3-immunized/infected mice, exhibited a 2–3-fold lower level of antigen- and *Tc*-specific proliferation and activation of CD8^+^T cells that was associated with mixed type1/type 2 cytokine response (Fig. 5, Table 2). Taken together, these results suggested that TcVac3 (± cytokine adjuvants) provided the host a capacity to respond to *T. cruzi* infection by induction of a robust CD8^+^ T cell and type 1 dominated cytokine responses.

### The Proinflammatory Type 1 CD8^+^ T Cell Response Subsided in Chronic Phase

**Figure 6:**
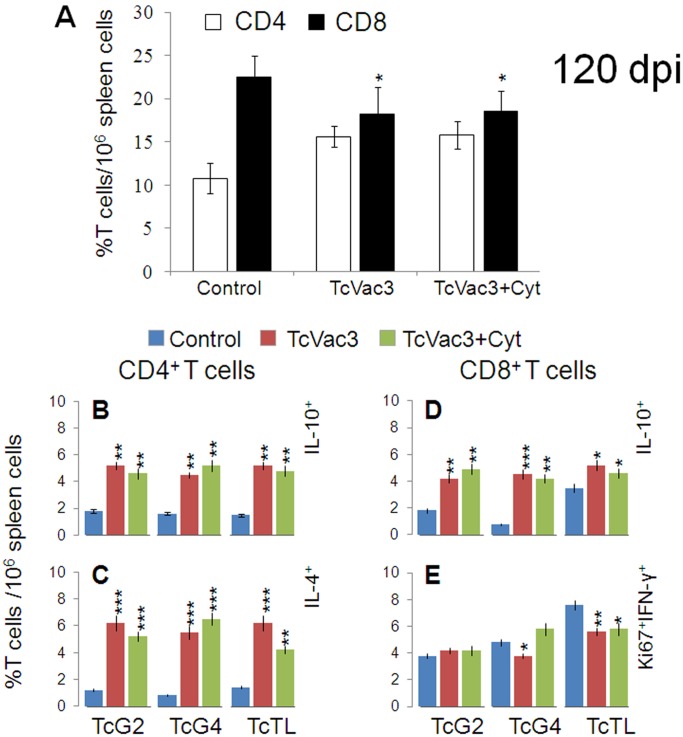
Functional profile of vaccine-induced T cells during chronic chagasic disease. Mice were vaccinated and infected as in Fig. 5, and harvested at day 120 post-infection. (**A**) Splenic frequency of CD4^+^ and CD8^+^ T cells. (**B &C**) Splenocytes were stimulated as in Fig. 5. (**B&E**) Shown are percentages of CD4^+^T cells that were IL-10^+^ (B) and IL-4^+^ (C); and the percentages of CD8^+^T cells that were IL-10^+^ (D) or Ki67^+^IFN-γ^+^ (E), measured by flow cytometry.

With progression to chronic phase (day 120 pi), the frequency of CD4^+^T cells remained at 10–16% of total T cells in all mice (Fig. 6), while frequency of CD8^+^T cells was lowered in vaccinated mice as compared to the controls (Fig. 6A). When *in vitro* stimulated with recombinant antigens or TcTL, CD4^+^ splenocytes of immunized mice exhibited a IL-10^+^ (range: 4–6%) and IL-4^+^ (range: 5–7%) phenotype (Fig. 6B&C), with no detection of IFN-γ^+^ or TNF-α^+^ phenotype (data not shown). The CD8^+^T cells of chronically-infected/immunized mice exhibited an emergence of IL-10^+^ (4–6%, Fig. 6D) phenotype and up to 70% decline in Ki67^+^IFN-γ^+^ phenotype when compared to that noted for CD8^+^T cells from the vaccinated mice in acute infection phase (compare Fig. 6E with Fig. 5C). The predominance of anti-inflammatory/immuno-regulatory cytokine profile in TcVac3-immunized mice during chronic phase was also evidenced by cytokine release. We noted a 3–8-fold decline in IFN-γ and TNF-α; and 3–5-fold increase in IL-4 and IL-10 production in antigen- and TcTL-stimulated splenocytes of TcVac3-immunized/chronically-infected mice when compared to that noted in splenocytes of vaccinated/acutely-infected mice (Table 1). Non-vaccinated/chronically-infected control mice continued to exhibit a substantial CD8^+^T cell proliferation with predominance of proinflammatory phenotype evidenced by *in vivo* cytokine profile and splenic cytokine release (Fig. 6D&E, Table 1, Table 2). These data (along with the data presented in Fig. 5) showed that TcVac3-primed proinflammatory T cell response was significantly expanded in response to challenge infection; and with progress to chronic phase, the proinflammatory T cells subsided, and a predominance of immunoregulatory/healing type T cell response emerged in vaccinated/chronic mice.

### Parasite Persistence and Chronic Tissue Damage were Arrested in Vaccinated Mice

**Figure 7:**
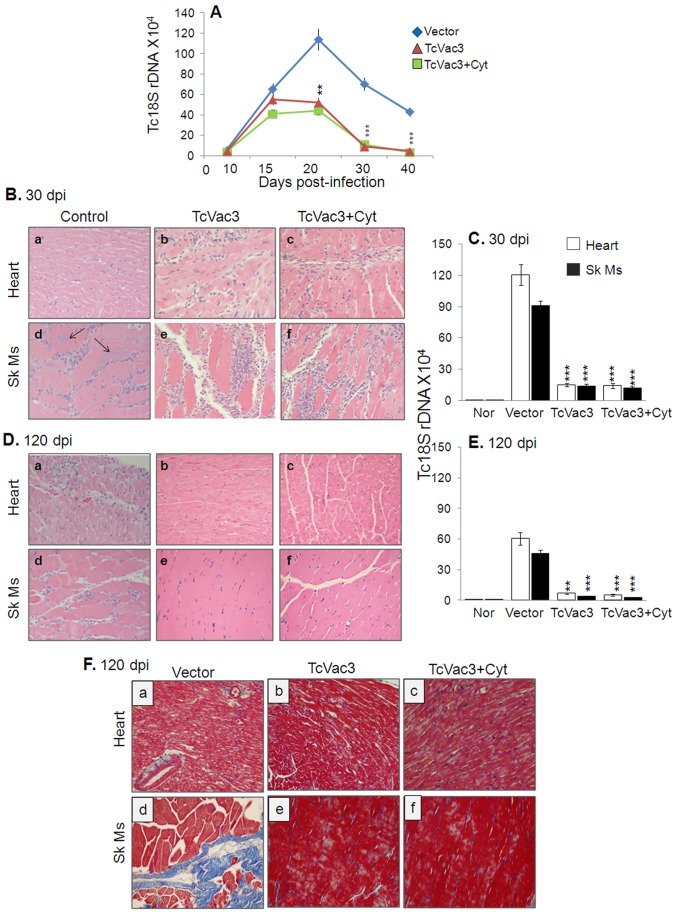
TcVac3 efficacy in controlling the inflammatory infiltrate, tissue pathology and parasite burden in mice. Mice were vaccinated and infected with *T. cruzi*. Peripheral blood collected at days 10–40 post-infection (A), and tissues harvested at day 30 (B&C) and 120 (D–F) post-infection were utilized. (**A**) Blood parasite level determined by real time PCR amplification of *Tc*18SrDNA sequence, normalized to *GAPDH.* (**B&D**) The representative images of H&E staining (blue: nuclear, pink: muscle/cytoplasm/keratin) of heart tissue and skeletal muscle sections (magnification: 20X) are shown (arrows indicate parasite nests). (**C&E**) Tissue parasite burden was determined by real time PCR amplification of *Tc*18SrDNA sequence, normalized to *GAPDH*. (**F**) Masson’s Trichrome staining (magnification 20X). The intense blue color shows collagen deposition (fibrotic area).

Detectable blood parasitemia that peaked during day 15–20 pi was noted in all infected mice (Fig. 7A). Mice vaccinated with TcVac3 (±cytokine adjuvants) exhibited 84–87% and 96–99% decline in parasitemia at day 30 and 40 post-infection, respectively, when compared to that noted in non-vaccinated/infected mice (p<0.001) (Fig. 7A). Histological analysis revealed extensive infiltration of inflammatory cells in the heart and skeletal muscle of all acutely-infected mice (Fig. 7B; Fig. S1). In agreement with enhanced activation of B and T cell responses, the infiltration of inflammatory cells in heart tissue (Fig. 7B.b&c) and skeletal muscle (Fig. 7B.e&f) of vaccinated/acutely-infected mice was extensive with either coalescing of inflammatory foci or diffused inflammation (histological score: 3–4; inflammation index 62–76%) as compared to that detected in non-vaccinated/acutely-infected mice (Fig. 7B.a&d) which showed mild to moderate inflammation (histological score 1–2; inflammation index 25–32%). Further, tissue parasite foci in skeletal muscle were remarkably reduced (0–2 per microscopic field, mf) in vaccinated mice harvested at day 30 pi as compared to control mice (2–6 parasite nests/mf, Fig. 7B.d–f). Real time PCR verified the above observations; we noted 88–90% and 86–88% reduction in parasite-specific *Tc*18SrDNA signal in heart and skeletal muscle of vaccinated/acutely-infected mice when compared to that detected in acutely-infected control mice (Fig. 7C, p<0.001).

All control and vaccinated mice survived *T. cruzi* challenge infection up to the chronic phase (120 dpi). During the chronic phase, infiltration of inflammatory cells was extensively reduced in the heart tissue (Fig. 7D.b&c) and skeletal muscle (Fig. 7.D.e&f) of TcVac3-immunized mice (histological score: 0–1; inflammation index: 2–8%, Fig. S1). In non-vaccinated/infected mice, chronic inflammation with diffused inflammatory foci (histological score: 3–4; inflammation index: 18–25%) was observed throughout the heart and skeletal muscle tissue (Fig. 7.D.a&d, Fig. S1). The chronic persistence of *T. cruzi*, determined by real time PCR analysis of *Tc*18SrDNA signal, was decreased by 92–94% and 88–90% in heart tissue and skeletal muscle, respectively, of TcVac3-immunized mice as compared to that noted in controls (Fig. 7E). Masson’s-Trichrome staining of tissues revealed significantly reduced fibrosis in vaccinated/chronically-infected mice with up to 94–96% reduction in the fibrotic area in the skeletal muscle and heart tissue as compared to the chronically-infected control mice (Fig. 7F.b,c,e,f; Fig. S1). Noticeable larger blue-stained areas, indicator of fibrosis, were evident in heart and skeletal muscle sections of chronically-infected control mice (Fig. 7F.a&d, Fig. S1). These results demonstrate that vaccination was effective in controlling the tissue parasite burden, and consequently, the associated immunopathology and fibrosis in chronic chagasic mice; while in the absence of vaccination, parasite persistence and immunopathology dominated.

## Discussion

In the present work, we studied the protective immune responses elicited by TcVac3 vaccine against *T. cruzi* infection and Chagas disease in C57BL/6 mouse model. The mouse-parasite model that we have utilized in our vaccine studies recapitulates the human development of chronic disease [24]–[25]. Infection of C57BL/6 mice with SylvioX10 (10.000 parasites/mouse) does not result in lethality, i.e., all mice survive infection, but develop chronic pathology. For this reason, we focus more on monitoring the pathology in chronic heart.

Studies using MVA as a delivery vehicle have indicated that this vector is particularly efficient when used as a booster vaccine. MVA itself can act as an adjuvant since it provides a signal to the innate immune system for its recognition and can boost both CD4^+^ and CD8^+^ T cells [26]–[28]. TcVac3 (consisting TcG2 and TcG4 candidate antigens) immunized mice elicited a strong antigen- and *Tc-*specific antibody response. The levels of IgG2b and IgG1 antibody isotypes were significantly elevated in immunized mice, and the antibody response was primarily of the Th1 type with IgG2b/IgG1 ratios being >1 (Fig. 1). Others have shown IgG1 and IgG2b are the predominant isotypes in mice immunized with amastigote surface protein (ASP-2) and trans-sialidase antigens [29]–[30], while IgG2a was elicited by immunization with kinetoplastid membrane protein-11 in rodents [31]. The serum antibody levels are not as meaningful as the functional activities reflected by antibody avidity and lytic activity that are better indicators of antibodies’ ability to bind to and promote pathogen killing [32]. The IgG’s avidity is expected to be low after primary antigenic exposure but increase progressively during subsequent weeks and months due to affinity maturation and antigen-driven B-cell selection. In our study, antibodies elicited by TcVac3 exhibited 50–80% antigen-binding avidity index and 92–96% trypanolytic efficiency, thus, suggesting that vaccinated mice were primed to respond with *Tc*-specific effector antibodies. Indeed, challenge infection resulted in a rapid and potent expansion of antibody response in TcVac3-immunized mice (Fig. 4). However, we observed the *Tc*- and antigen-specific avidity index of immunoglobulins in vaccinated mice was decreased by 30–32% and 12–15%, respectively. One explanation for the observed decline in avidity of antibodies during acute infection is that the antibodies with high avidity were bound to remove the antigen *(T. cruzi),* and, therefore, consumed. This notion is supported by our observation in reduction (up to 90%) of acute parasite burden in TcVac3-immunized mice as compared to what was noted in infected controls (Fig. 7). The observation of a persistent antibody response in TcVac3-immunized mice at the onset of chronic phase when most of the parasites were removed is puzzling, but also noted in human patients. A high abundance of anti-*Tc* lytic antibodies in chronic patients in indeterminate phase as compared to those exhibiting clinical disease is suggested to indicate the protective role of lytic antibodies [33]. Further, antibody persistence in patients treated with anti-parasite drugs that exhibit negative hemo-cultures for over ten years is also noted [34]. There is no consensus on whether or not consistent B cell maturation and antibody secretion indicates antigen (*Tc*) persistence [35]–[36] or persistence of antibodies indicates long-term surveillance ensuring the host is free of pathogen. Further studies will be required to determine the mechanisms of antibody persistence long after control of parasite burden as we have observed in vaccinated/infected mice. Yet, our data clearly establish that TcVac3 elicited high avidity, lytic antibody response via successful priming of the B cells was effective in controlling challenge *T. cruzi* infection.

A recent review of literature on human T cell immunity indicates a decreased frequency of T cells in acutely-infected patients either due to a temporarily suppressed or exhausted response both of which favor *Tc* survival [37]. Studies in murine models of *T. cruzi* infection suggest pr-inflammatory cytokine-secreting CD4^+^ and CD8^+^ T cells, and cytotoxic T lymphocytes activity are associated with protection from infection, while over production of anti-inflammatory cytokines or blockage of pro-inflammatory cytokine production correlated with increased susceptibility to *T. cruzi* infection. In chronically-infected patients, a high frequency of inflammatory activated T cells, especially granzyme^+^CD8^+^T cells is invariably associated with pathology and tissue destruction in Chagas disease [36]–[39]. Likewise, several studies have demonstrated a correlation between production of inflammatory cytokines by CD4^+^T cells with clinical disease and the production of IL-10 with clinically asymptomatic state in chronic chagasic patients [38]–[39]. We, therefore, performed a detailed analysis of the cellular immune responses to determine if TcVac3-induced T cell responses were protective against *T. cruzi* infection or were modulated as the host entered the chronic phase. Our data showed that TcVac3 primed CD4^+^ and CD8^+^ T cells that prolifically expanded in response to *in vitro* or *in vivo* antigenic stimulation. Overall, CD8^+^T cells were primed and expanded more robustly (as compared to CD4^+^T cells), and were IFN-γ or TNF-α producers and exhibited a cytolytic effector phenotype evidenced by increased expression of CD107a, perforin, and IFN-γ. Though CD4^+^T cell numbers were not increased *in vivo* upon challenge infection of vaccinated mice, IFN-γ producing CD4^+^T cells likely assisted the phagocytes in parasite killing, and served as helper cells for the development of CD8^+^ CTL effector cells [40]–[41]. Importantly, the proliferation and expansion of Th1 1 effector CD8^+^T cells correlated with a significant control of *T. cruzi*, evidenced by the observation of >6-fold less parasite burden in TcVac3-immunized/infected mice as compared to that noted in control mice at day 30 pi. Overall, our observations provide strong evidence that a dominant recall and proliferation of TcVac3-primed CD8^+^T cells provided early control of *Tc* replication in mice.

In chronic phase (120 dpi) persistence of inflammatory response constituted of IFN-γ^+^ and TNF-α^+^ T cells associated with tissue fibrosis and cell death were noted in infected control mice. We, however, found that infiltration of inflammatory infiltrate, parasite persistence, and fibrotic plaques were virtually undetectable in heart and skeletal muscle of TcVac3-immunized/chronically-infected mice. The control of inflammation was evidenced by >90% reduction in the splenic frequency of proinflammatory T cells and an emergence of IL-10^+^T cells in TcVac3-immunized mice during the chronic phase (120 dpi). In human chagasic patients, gene expression profiling of myocardial tissue from chagasic patients have shown that 15% of the genes known to be selectively up regulated were IFN-γ-inducible [42], and high levels of TNF-α has been associated with worsened cardiac function in chagasic patients [43]. These observations point to the pathologic significance of IFN-γ and TNF-α in human chagasic cardiomyopathy. Notably, the IL-10^+^ regulatory T cells are predominantly found in clinically-asymptomatic chagasic patients [44], indicating that these cells play an important role in regulation of pathogenic responses in chronic patients. Our data presented in this study provide strong evidence that TcVac3-induced protection from *T. cruzi* infection and Chagas disease was associated with IL-10-dependent regulatory networks critical for arresting the clinical evolution of disease; to be further delineated in future studies.

In deciding which of our strategies might be more efficacious as a clinical vaccine it is important that the vaccine formulation elicits potent protective immune response, is safe and simple. Summarizing our efforts, we have found that TcG2 and TcG4 elicited different levels of B and T cell immunity against *T. cruzi* depending upon the mode of delivery (DNA/DNA<DNA/protein = DNA/MVA), and co-delivery of antigens elicited additive immunity that was achieved by individual candidate antigens. While delivery of antigens as a DNA/protein vaccine provided similar level of protection as was observed with DNA/MVA approach in this study; however, DNA/protein vaccine was more complex requiring three antigens (TcG1, TcG2, and TcG4) and cytokine adjuvants (IL-12- and GM-CSF) (total six plasmids) followed by purification of recombinant proteins (three proteins). Further, protective efficacy (∼90% reduction in *T. cruzi* burden) was achieved when we delivered four doses of this vaccine [11]. These studies also suggested that immunization with IL-12 and GM-CSF encoding cytokines alone before challenge infection can moderately enhance the parasite-specific Th1 immune response; however, this response was not sufficient to control parasite burden and provide protection from *T. cruzi*-induced pathology.

In comparison, TcVac3 vaccine was constituted of only two antigens (TcG2 and TcG4) that were delivered by DNA/MVA approach, and addition of IL-12 and GM-CSF cytokine adjuvants was not beneficial as these cytokines did not enhance the vaccine-primed B and T cell responses. Further, the two-component TcVac3 vaccine provided potent resistance to *T. cruzi* infection, and importantly, resulted in a dominance of IL-10-dependent immunoregulatory response, suggested to be an indicator of clinically asymptomatic state in chronic phase. Recombinant MVA (rMVA) expressing immunogens from a variety of infectious agents (e.g., HIV, *Plasmodium falciparum*) or tumor-associated antigens have been successfully tested in phase I and phase II clinical trials [45]–[48], and our data suggest that TcVac3 has a potential to enter clinical trials designed for testing vaccine efficacy against *T. cruzi* infection.

In summary, we have demonstrated that TcVac3 constituted of TcG2 and TcG4 antigens delivered by a DNA-prime/MVA-boost approach enabled the host to control acute *T. cruzi* infection, and subsequently, prevent the persistent activation and infiltration of inflammatory cells in the heart that otherwise occur due to parasite persistence and result in tissue destruction during the chronic phase. MVA (single dose) or MVA-prime/MVA-boost will be tested in an effort to simplify the vaccine composition, especially if MVA-MVA approach would result in similar level of protection as is observed with two-component DNA-prime/MVA-boost vaccine in this study.

## Supporting Information

Figure S1 **Shown is the quantitative scoring of inflammation from H&E-stained tissue sections at 30 (A) and 120 (B) days post-infection and fibrosis (C) i.e. the percentage of Masson’s Trichome stained collagen area at 120 dpi.**
(TIF)
