# Supplementary figures and images for: TcVac3 Induced Control of Trypanosoma cruzi Infection and Chronic Myocarditis in Mice
Source: PLoS One. 2013 Mar 26;8(3):e59434. doi: 10.1371/journal.pone.0059434 (PMC3608676; doi:10.1371/journal.pone.0059434)

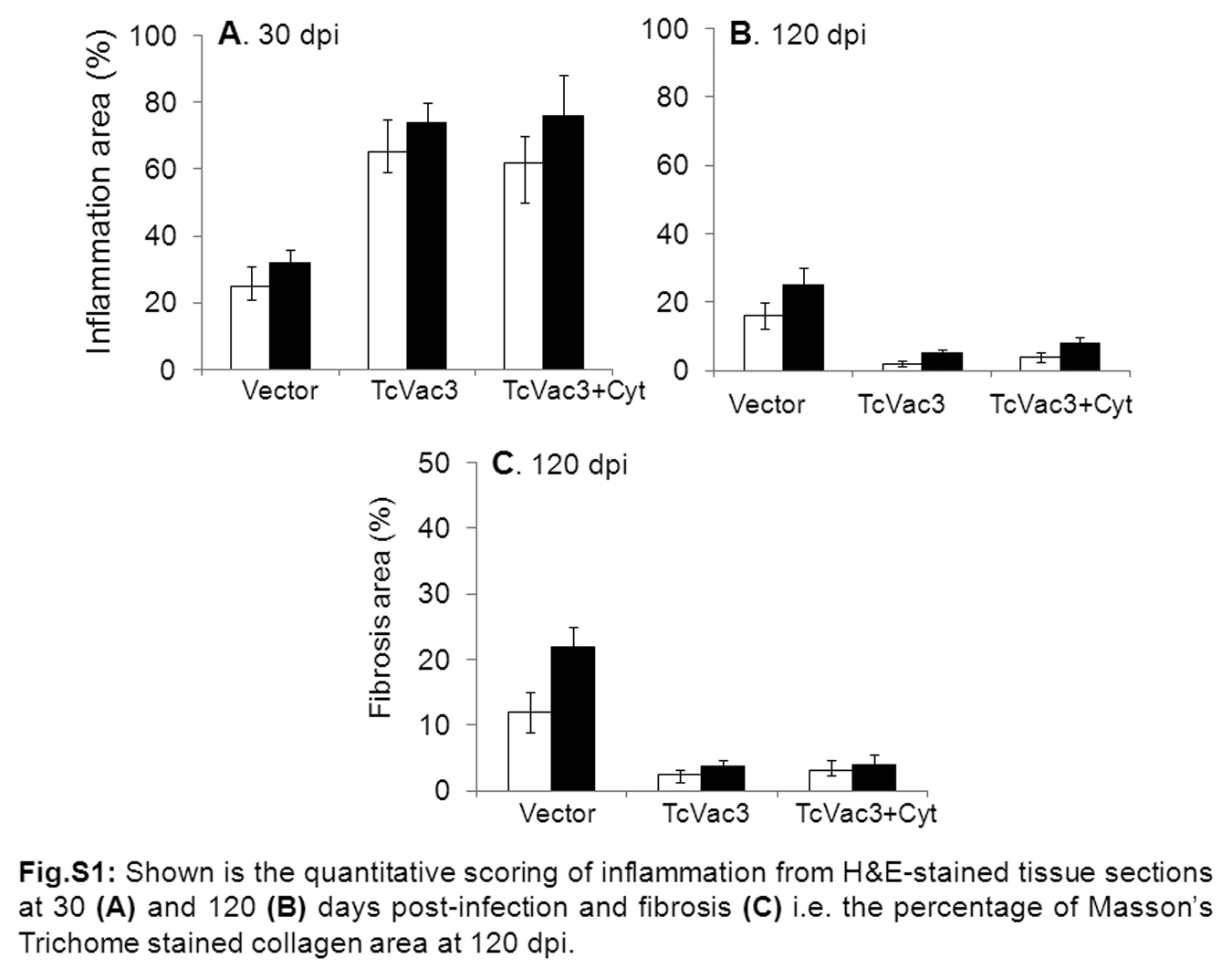

Supplement: Figure S1 — Shown is the quantitative scoring of inflammation from H&E-stained tissue sections at 30 (A) and 120 (B) days post-infection and fibrosis (C) i.e. the percentage of Masson’s Trichome stained collagen area at 120 dpi. (TIF) [file pone.0059434.s001.tif]
